# Recent 3D Cell Culture Models: From Biomedical Applications to Machine Learning

**DOI:** 10.3390/biomedicines14020379

**Published:** 2026-02-06

**Authors:** Agnieszka Rusak, Benita Wiatrak

**Affiliations:** 1 Division of Histology and Embryology, Department of Human Morphology and Embryology, Faculty of Medicine, Wroclaw Medical University, T. Chalubinskiego 6a St., 50-368 Wroclaw, Poland; 2 Department of Pharmacology, Faculty of Medicine, Wroclaw Medical University, J. Mikulicza-Radeckiego 2 St., 50-345 Wroclaw, Poland; benita.wiatrak@umw.edu.pl

The in vitro cell culture is one of the most widely used experimental method for modelling physiology and pathology—from drug screening and mechanistic studies to assessing biomaterials’ biocompatibility. The establishment of the first human cell line, HeLa, derived from a biopsy of uterine cancer taken from Henrietta Lacks in 1951 [1], marked the turning point for cancer cell culture in monolayer conditions. For decades, the in vitro culture of cancer or normal cells in monolayers has preceded in vivo experiments and provided fundamental findings for a wide range of medical applications, and will continue to do so. However, decades of intensive use of monolayer cultures have also revealed persistent limitations of conventional 2D systems, including reduced tissue-like architecture, altered polarity, and the lack of physiologically relevant gradients and extracellular matrix (ECM) cues. For a few years now, cell culture in 3D models such as spheroids, scaffolds and organoids has been considered a more relevant in vitro model due to cell-to-cell interactions and cross-talk in the microenvironment, which provide a much more advanced response to drug uptake systems, new targeted therapies, physiological processes and interactions with biomaterials [2,3].

In this Special Issue, we focus on new 3D cell culture models in a broad range of biomedical scientific areas that may inspire researchers when designing experiments. The articles in this Special Issue all have the potential to contribute to new targeted therapies or biomedical engineering, and they all demonstrate interdisciplinarity, nature of modern 3D research, bridging cell and molecular biology, materials science, bioengineering, and data-driven analysis.

The physiology of the mammary gland was presented by Chen and colleagues using 3D mammary cultures [4]. In this paper, the authors present evidence that immunological conditions influence lactation. Mammary acini from mice were cultured on Matrigel, stimulated with prolactin, and exposed to IFN-γ and TNF-α. The authors observed that these cytokines attenuated prolactin signalling by decreasing the phosphorylation of STAT5. Consequently, β-casein expression decreased and acinar architecture was altered. These findings suggest that a high level of these cytokines, as occurs during infection, for example, may block lactation. Based on these results, Chen and his colleagues also demonstrated that cytokine stimulation caused iNOS expression. However, iNOS inhibition led to improved lactation and a normalised acinar structure. This work may be clinically useful in cases of inflammation disorders co-existing with lactation problems.

Ene and colleagues introduced a choroid plexus organoid model [5] used to generate extracellular vesicles (EVs) that were subsequently loaded with curcumin. The authors present a model of organoids in which extracellular vesicles (EVs) were isolated from the medium and loaded with curcumin. The authors then exposed the choroid plexus organoids to Amyloid Beta (Aβ) 42 Oligomer, a factor involved in the pathophysiology of Alzheimer’s disease, as well as to EVs with curcumin. The results indicate that fresh EVs with curcumin increased the level of TNF-α, but lyophilisation decreased the levels of TNF-α and IL-6. These observations suggests that lyophilised EVs with curcumin may have a protective effect against Aβ42-induced neuroinflammation in Alzheimer’s disease, supporting their potential as a cell-free therapeutic strategy.

Chiabotto and colleagues addressed hepatic fibrosis using 3D liver spheroids [6]. Presented the role of the therapeutic effect of extracellular vesicles (EVs) derived from human mesenchymal stromal cells (MSCs) on spheroids consisting of HepG2 hepatocytes or primary hepatocytes mixed with hepatic stellate cells (HSCs) [6]. In this paper, the authors showed that EVs from MSCs decreased the adverse fibrotic effects of treatment with TGF-β1. Treatment with EVs decreased the expression of collagen αSMA, a main marker of liver fibrosis. This effect of EVs derived from MSCs may be crucial in the biological treatment of liver fibrosis.

Gdesz-Birula and colleagues developed bone marrow stromal spheroids composed of HS-5 mesenchymal stromal cells and investigated leukaemia cell adhesion under hypoxic conditions [7]. Using OCI-AML3 acute myeloid leukaemia cells and optical tweezers to assess adhesion in real time, they tested sonidegib—a Hedgehog pathway inhibitor approved for basal cell carcinoma. The authors reported that sonidegib reduced OCI-AML3 adhesion to stromal spheroids by inhibiting Hedgehog pathway activity. This study highlights how 3D niche-mimicking systems, combined with sensitive biophysical measurements, can help identify therapeutic strategies aimed at disrupting tumour–microenvironment interactions.

In one paper in this Special Issue, artificial intelligence (AI) was employed to analyse histological cross-section images of hydrogel filaments [8]. Using the freely available Google platform ‘Teachable Machine’ to evaluate over six hundred images of cross-sections of a combination of sodium alginate and gelatin polymers after haematoxylin and eosin staining [8]. This work illustrates how accessible machine learning solutions can support standardised image-based classification and may be adapted for broader histological and biomaterials applications. In turn, the role of trans-retinoid acids was investigated by Higashide and colleagues using monolayer and spheroid models of human ARPE19 retinoid cells in normoxia and hypoxic conditions [9]. In these studies, the authors revealed that trans-retinoid acids change the cellular state from mitochondrial respiration to glycolysis under hypoxic conditions, but do not affect ROS levels. This effect was enhanced by TGF-β2. Cells’ stiffness changed in hypoxia and TGF-β2 treatment, and the effect of these two factors acting synergistically was observed in both 2D and 3D models, although retinoid acids played an important role. These results may shed light on the role of TGF-β2 and hypoxia, which are main factors connected with retinal pathology in the context of vitamin A presence.

In this Special Issue, we also present a paper by Dobrzyński and his colleagues on a 3D biomaterial for jawbone osteosynthesis and regeneration [10]. A titanium–aluminium–vanadium (Ti-6Al-4V) alloy, which is a biocompatible material for implants, was 3D-printed using CAD technology. This method allows the design of shapes for individual patients, as well as providing structural and surface modifications. In this paper, the authors observed favourable material surface properties for the adhesion of human osteoblasts (hFOB1.19), no cytotoxicity of the analysed material on L929 and Balb/3T3 cells, and a reduction in microbial biofilm formation using strains of Lactobacillus rhamnosus, Staphylococcus epidermidis, Streptococcus mutans and Candida albicans. These results indicate that implants obtained using the 3D CAD method show promise in stomatological implantology.

Taken together, the contributions in this Special Issue underscore the value of 3D culture systems as versatile platforms to model tissue-level processes, interrogate disease mechanisms, and evaluate therapeutics and biomaterials in a more physiologically relevant context. They also highlight an emerging methodological direction: as 3D models yield richer and more complex datasets, machine learning and other computational approaches will be essential for reproducible quantification, automated image analysis, and cross-study comparability. Further advances in standardisation, readout harmonisation, and data-driven analytics will help unlock the full translational potential of 3D models across biomedical research.
